# Engaging New Migrants in Infectious Disease Screening: A Qualitative Semi-Structured Interview Study of UK Migrant Community Health-Care Leads

**DOI:** 10.1371/journal.pone.0108261

**Published:** 2014-10-15

**Authors:** Farah Seedat, Sally Hargreaves, Jonathan S. Friedland

**Affiliations:** International Health Unit, Section of Infectious Diseases and Immunity, Commonwealth Building, Hammersmith Campus, Imperial College London, London, United Kingdom; Kilifi, Kenya

## Abstract

Migration to Europe - and in particular the UK - has risen dramatically in the past decades, with implications for public health services. Migrants have increased vulnerability to infectious diseases (70% of TB cases and 60% HIV cases are in migrants) and face multiple barriers to healthcare. There is currently considerable debate as to the optimum approach to infectious disease screening in this often hard-to-reach group, and an urgent need for innovative approaches. Little research has focused on the specific experience of new migrants, nor sought their views on ways forward. We undertook a qualitative semi-structured interview study of migrant community health-care leads representing dominant new migrant groups in London, UK, to explore their views around barriers to screening, acceptability of screening, and innovative approaches to screening for four key diseases (HIV, TB, hepatitis B, and hepatitis C). Participants unanimously agreed that current screening models are not perceived to be widely accessible to new migrant communities. Dominant barriers that discourage uptake of screening include disease-related stigma present in their own communities and services being perceived as non-migrant friendly. New migrants are likely to be disproportionately affected by these barriers, with implications for health status. Screening is certainly acceptable to new migrants, however, services need to be developed to become more community-based, proactive, and to work more closely with community organisations; findings that mirror the views of migrants and health-care providers in Europe and internationally. Awareness raising about the benefits of screening within new migrant communities is critical. One innovative approach proposed by participants is a community-based package of health screening combining all key diseases into one general health check-up, to lessen the associated stigma. Further research is needed to develop evidence-based community-focused screening models - drawing on models of best practice from other countries receiving high numbers of migrants.

## Introduction

With an estimated 72.6 million migrants now residing in Europe, the region is an increasingly important recipient of approximately one third of the international migrant population [1], [2]. In the past decade the UK in particular has received a sizeable and increasing number of new migrants, which has had important implications for public health services [3], [4]. Infectious diseases are believed to be the key health issue for new migrants from high-prevalence countries, with asylum seekers and refugees considered to be particularly affected [5]. Migrants bear the largest burden of infectious disease in the UK; approximately 70% of newly diagnosed UK tuberculosis (TB) and 60% of new HIV cases are in migrants, with comparable trends expected for hepatitis B and C [3], [5]–[7]. London now has the highest tuberculosis rate among all capital cities in western Europe [8]. Numerous factors contribute to the vulnerability of new migrants to infectious diseases, with migrants - and ethnic minorities more broadly - known to face barriers to healthcare, which may result in delays to screening and diagnosis [4], [9]. Tackling infectious diseases may raise specific issues, including stigma and fear of discrimination [10]–[12]. New migrants may be particularly affected in terms of their ability to access and benefit from screening programmes for infectious disease as they attempt to navigate a new health system [13], [14].

How best to screen new migrants, and what to screen for, remains an ongoing debate in the UK and Europe [15]–[20], with approaches varying considerably [1], [21], [22]. The UK Government, for example, has recently closed down its port of entry tuberculosis screening because of concerns that it was poorly run, discriminatory, and not cost-effective, opting instead for pre-entry screening. Evidence suggests that it is critical to engage new migrants from high-prevalence countries early on if they are planning to reside in the UK for a period of time. TB is known to surface 3–5 years after arrival [6] and data suggests that HIV is often acquired in a migrants' home country with most unaware of their status on arrival to the UK [23]. The issue of vaccination for infectious diseases may also be important for health-care providers to address. There have been calls to strengthen primary-care-based screening programmes, and to place renewed focus on latent tuberculosis screening to tackle the rising tide of tuberculosis [17], [18]. However, there remains a paucity of data on barriers to, and acceptability of, screening programmes for infectious diseases specifically in newly arrived migrants, and potential ways forward; addressing these shortfalls remains an important component in the strategy to tackle rising rates of infectious diseases.

We did a qualitative semi-structured interview study of migrant community health-care leads, who represent dominant new migrant groups in London, UK. The aim was to explore their views around barriers, accessibility, and acceptability of screening for HIV, tuberculosis, hepatitis B, and hepatitis C.

## Methods

We did a series of semi-structured face-to-face interviews to explore migrant community health-care leads' perceptions about (i) the barriers to screening for HIV, TB, and hepatitis B and C faced by new migrants; (ii) acceptability of screening; and (iii) innovative approaches to improve screening uptake in new migrants. We defined new migrants as foreign-born individuals who had resided in the UK for less than 5 years, arriving from countries outside Western Europe, North America, Australia, and New Zealand. Therefore, we sought information about new migrant groups from high prevalence disease countries. We carried out and reported this study using COREQ guidelines [24] as well as the quality guidelines of Mays and Pope (2000) [25]. The study was approved by the Imperial College Research Ethics Committee.

### Participant selection and recruitment

We approached migrant community health-care leads who encompassed dominant groups of new migrants in the study site – which was a high migrant area of West London (Hammersmith and Fulham) where 42.8% of residents defined as foreign born [26]. The inclusion criteria were community leads who were expert professionals with a working knowledge of the health needs of new migrants within the communities and nationality groups they represented, who were >18 years of age, and capable of giving informed consent. This approach – of recruiting community leads rather than the new migrants themselves - is one used successfully elsewhere, and was repeated with the aim of acquiring an overview of the issues facing new migrants across a broad range of nationality groups [27].

**Figure 1:**
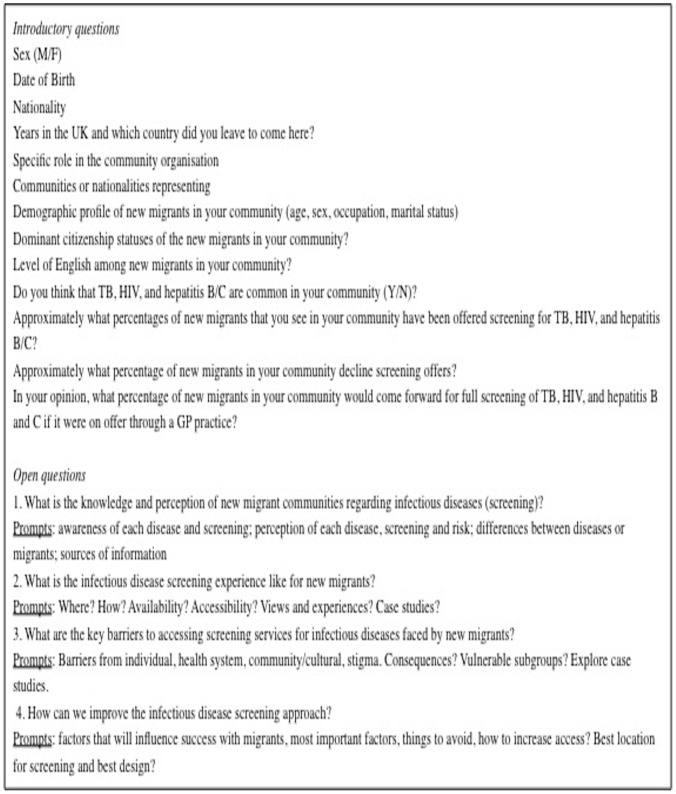
Interview topic guide.

We recruited participants using purposive sampling to enable exploration of particular aspects of behaviours relevant to the research questions. We drew up a sampling frame for the target population by carrying out internet searches of London-based community groups around the study site. This list was used as a starting point to generate a list of relevant individuals working within these community groups who would meet study inclusion criteria. Potential participants were then directly approached by telephone and invited to participate. Those who were interested in participating were emailed a Participant Information Leaflet about the study, and re-contacted to confirm participation and arrange the interview. Purposive sampling allowed us to then use our initial participants to establish subsequent contact with other relevant participants. Purposive sampling also allowed us to recruit a mix of nationality groups to represent the major new migrant community groups. We developed a topic guide (Figure 1) of both structured and open questions based on previous work conducted by the authors in collaboration with community leaders in another London-base study site [27] and pilot tested it on the first participant. Participant recruitment continued until data saturation was achieved for all categories.

### Data collection and analysis

In all but two cases, FS (female, British Asian) conducted interviews at participants' workplace in a private room where only the interviewer and participant were present. FS had previously been trained to conduct research interviews in the field of migrant health. After acquiring written informed consent, participants were reminded of the study aims and assured that all information they shared would be confidential and presented in an anonymous format, and the interview then commenced for 30–90 minutes.

The interviews were audio-recorded with permission (Sony VOR Microcassette Recorder M-740V) and transcribed verbatim for analysis by independent transcribers (anonymously) after each interview. Case memos were made after every interview in addition to theoretical memos to assist in the formulation of theories. Data were analysed using the principles of grounded theory, which involved systematically collecting and analysing data simultaneously throughout the research process. Data were managed using QSR NVivo 10 software.

Data was first coded by open coding to generate concepts. After carrying out the first three interviews a list of all the codes elicited from the transcripts were grouped into a list of categories using axial coding. To guard against selectivity, two researchers (FS/SH) independently conducted these processes and discussed the initial interpretations of the data, the reliability of the codes, explanations of particular codes, and additional areas for exploration within the subsequent interviews. This discussion combined with the research questions that shaped the topic guide formed the basis of the coding framework thereon. Transparency in the method and regular checks and discussion about the codes and categories mitigated experimenter bias.

A constant comparison approach was then used whereby transcripts were reviewed and codes were developed in an iterative process. By constantly comparing the codes and themes and using deviant case analysis to explore conflicting views, we were able to interpret the data with validity. As interviews were completed, selective coding was conducted where the most common codes and those seen as most revealing about the data were emphasised and unified around a core framework. A final re-check of all transcripts was carried out to check whether all text had been accounted for and to ensure that all initial open-codes were incorporated into each theme where appropriate. Member checking was also adopted by sending the final report to all the participants in the study for feedback.

## Results

### Sample characteristics

50 organisations working with migrants on health issues in the survey site were identified from the initial internet search, of which 34 were unable to support us in identifying participants within their own organisation (2 shut down; 5 contact details were out of date; 19 did not respond; 4 could not identify potential participants within their own organisation, 4 organisations declined). Of the four organisations that declined participation: one only dealt with more settled migrants who had been in the UK for long periods of time; two stated that they did not have a health-care lead for an interview; and one declined due to lack of experience in the field of health care specifically.

From the initial contact with the remaining 16 organisations, 20 community health leads were identified and agreed to be interviewed. By interview 20, data saturation was achieved, no new or relevant material arose, and it was highly probably that additional interviews would not have influenced results. There were an equal number of male and female participants (mean age 42.7 years). Nine participants worked in HIV & AIDS related organisations, one in a TB related organisation, and one in a hepatitis B related organisation; 16 of 20 were migrants themselves and of the remaining, 3 out of 4 were from ethnic minority groups in the UK. Five participants were Chief Executives/Directors of the organisations they represented, 5 were programme coordinators, 2 were project leaders, 3 were project managers, 2 were community development officers, 1 was a project officer, 1 was a volunteer, and 1 was a faith engager. Participants represented new migrant communities from 39 diverse nationalities across Africa, Americas, Asia and Europe (age range in these communities 20–85 years). The majority of new migrants represented were refugees and asylum seekers, followed by migrants claiming citizenship, and those on student visas; most of the population under discussion, therefore, were considered by participants to be of low socioeconomic status (Table 1).

**Table 1:** Characteristics of participants and the new migrants communities they represent.

Characteristics	*N*
Participants	20
Gender of participants	F (10), M (10)
Mean Age (range)	42.77 (25–64 years)
Mean years in the UK (range)	21.59 (4–43 years); 4 born in the UK
Country of birth	Africa (8) – Kenya (1), Nigeria (3), Somalia (2), Uganda (1), Zambia (1)
	Asia (3) – Bangladesh (1), Iran (1), Malaysia (1)
	Europe (7) – Greece (1), Poland (1), Ukraine (1), United Kingdom (4)
	Americas (2) – Colombia (1), Jamaica (1)
Nationalities of new migrants represented*	Africa (13) – Algeria, Burkina Faso, Cameroon, Cote D'Ivoire, Egypt, Eritrea, Ethiopia, Ghana, Kenya, Morocco, Nigeria, Senegal, Somalia, South Africa, Tunisia, Uganda, Zambia, Zimbabwe,
	Americas (3) – Caribbean, Chile, Colombia, Ecuador, Venezuela,
	Asia (9) – Bangladesh, China, India, Iraq, Iran, Lebanon, Nepal, Pakistan, Philippines, Syria,
	Europe (3) – Latvia, Lithuania, Poland, Russia, Slovakia, Ukraine
Gender of new migrants represented	Majority female (10), Majority male (5), Equal (5)
Age range of new migrants represented	20–85 years old
Citizenship status of new migrants represented**	Claiming citizenship (11), EC citizen (5), Indefinite resident (2), Refugee or asylum seeker (14), Spouse visa (2), Student visa (10), Tourist (1) Undocumented (3), UK citizen (5), Work permit (5), Unknown (2)
Level of English of new migrants represented	A few words (9), Conversational (6), Fluent (5)

*Participants focussed on 39 different nationalities in total.

The numbers in brackets represent the number of participants who mentioned that the region is a majority that they represent.

**The numbers in brackets represent the number of participants who mention the status as one of the dominant statuses that they represent.

### Key themes

Two key themes emerged from the interviews:

- Existing screening models are not perceived to be widely accessible to the new migrant community. The main barriers that discourage use are disease-related stigma present in their own communities and services being perceived as non-migrant friendly. New migrants may be disproportionately affected and delays to screening may impact on health status.
- Screening is certainly acceptable to new migrants. Participants stressed the need for service providers to bring accessible and migrant-friendly screening into the community, strengthening collaborations with community-based organisations, and proposed a community-based package of health screening combining all of the diseases into one general health check-up with the aim of reducing stigma.

The barriers and facilitators described were points most commonly recommended or strongly recommended by participants.

### Screening is inaccessible to the new migrant community

Participants identified a range of barriers to screening for infectious diseases at the health system, community, and individual level (Table 2). There was strong agreement among participants that screening for infectious diseases was not accessible to the new migrant community in the UK. Most participants felt that although screening services do exist, they are not adequately reaching people in the community, stating that “people don't even know that these [screening services] exist” (Participant 11, age 31, male, Latin American community) and that new migrants specifically “do not easily take them up” (Participant 1, age 52, male, African community). Some participants added that the services are not well-publicised as information on them is not given to new migrants who are attempting to navigate a new health system, while one participant believed that services at present are “not pro-active” in encouraging new migrants to come forward for testing, and focus remains too much on the “treatment angle” rather than a preventative approach (Participant 13, age 32, male, migrant communities).

**Table 2:** Barriers to screening reported.

Level	Barrier
System and provider level barriers	Capacity/funding shortages for community organisations
	Lack of advocacy and promotion
	Lack of confidentiality
	Lack of psycho-social support services
	Low awareness of diseases amongst health professionals
	Poor link with community organisations
	Migrant unfriendly services
	Discrimination and stigma from health professionals
	Cultural insensitivity
	Inhospitality
	Time and distance to services
Community level barriers	Culture
	Cultural mentality and baggage
	Extra pressure for women regarding virginity and family role
	Lack of openness
	No prevention culture
	Faith, lack of openness, and stigma
	Language
	Stigma and misconceptions
Patient level barriers	Fear of a lack of confidentiality
	Fear of cost and eligibility (perceived or actual)
	Fear of disease status
	Isolation
	Lack of awareness and knowledge of diseases
	Lack of confidence using new health system
	Lack of screening services or health-system knowledge
	Misunderstanding between health system in current residence vs country of origin
	Low perception of risk
	Low priority on immigrant list
	Poorer health-seeking behaviour in men

The most cited barrier, highlighted by 19 of 20 participants, was the stigma and misconceptions that new migrant communities' hold about the key diseases. Participants identified that stigma within their own communities is the “biggest barrier to date” and the “biggest dilemma” they face when considering going for screening. Each disease has more than one different type of stigma in the different communities. According to participants, we found that TB, HIV, and hepatitis B and C can be perceived by new migrant communities as being “fatal” and/or “highly infectious”, which generates fear of testing. As a result of stigma, new migrants may “run-away” from the infected individuals, not inviting infected individuals to their houses, eating with them, or wanting to be near them. Participants said this discourages people from attending screening, because they may have to face such disease-related social consequences if people know they have attended screening and an infection is found. TB specifically is perceived in the Somali and Asian community as a “disease of the poor” and in the Asian community also as “hereditary”.

Participants unanimously considered HIV to still be the most stigmatised disease of the four. Issues around stigma for HIV are complex and deeply interlinked with a migrant's culture and faith. Stemming from cultural and religious beliefs, HIV is perceived as a result of a “sinful” and “immoral” lifestyle including “pre-marital sex”, “drug use”, “promiscuity”, or “being gay”. Participants said that new migrant communities see HIV as being self-inflicted, a “punishment from God” and a “well-deserved disease”. This prevents people from attending screening “as nobody wants to be seen in that way” (Participant 5, age 59, male, Somali community). Two participants also indicated that in the African migrant community, HIV is strongly associated with TB. Therefore, people often misconceive patients who have TB as having HIV or vice versa. Little was known about hepatitis stigma and only three participants indicated that the stigma could be similar to the way in which HIV is perceived, “filled with things about a lifestyle, moral behaviour and stereotypical things” (Participant 2, age 44, male, Ukrainian community).

The second most important barrier reported by participants was that the screening services are not “migrant friendly”. Twelve participants expressed concerns about the cultural insensitivity experienced by new migrants within services, where sometimes assumptions are made about the patients. In addition to cultural insensitivity, eight participants indicated that new migrants were frustrated with the inhospitable and unfriendly experiences when they accessed services. Many of these participants were concerned with the inhospitality of receptionist staff in particular; one participant said that this was the case even where receptionists were from an ethnic minority or migrant background themselves. In addition, seven participants identified discrimination from health-care professionals as an important barrier. Some participants mentioned that the discrimination was against the new migrants' country of origin, for example one participant who went to ask for results was asked by a nurse, “Is this the way you guys behave in Africa?” (Participant 13, male, age 32, all migrant communities), while another participant reported patients being asked: “why don't you speak English, how long have you been here?” (Participant 20, age 36, female, all migrant communities). Language barriers may be a particular issue for new migrants on arrival; most new migrants represented in this study spoke little English (Table 1). Another participant identified that it was a combination of their migrant status, as well as their infectious disease, that lead to discrimination. Four participants, however, felt that services were culturally sensitive and had not come across any cases or complaints from within their communities.

Participants also mentioned a number of barriers that may be unique to new migrants, including the issue of a lack of entitlement to free health care and confidentiality issues (table 2). Participants commented that new migrants “have to find their own way” (Participant 5, age59, male, Somali community), that “they don't know what exists…that they have the right, that it's free” (Participant 11, age 31, male, Latin American community). New migrants “may not think they're entitled to help here and they may not think they could just go and get it” (participant 15, age 48, male, African community). New migrants may also have confidentiality issues “concerns around immigration” and whether their “disease status will be shared with immigration services” (Participant 12, age 27, male, Afro-Caribbean community). Participants report that new migrants are concerned that clinical services, especially in hospital settings, are “government bodies and attached to the government…therefore somehow linked to immigration” (Participant 10, male, age 29, Asian community). In particular, some new migrants perhaps “don't have a visa” and are scared of “exposing themselves” by attending screening as “they will be deported because of their status” (Participant 18, age 59, female, African community).

### Barriers to care have implications for health status

**Figure 2:**
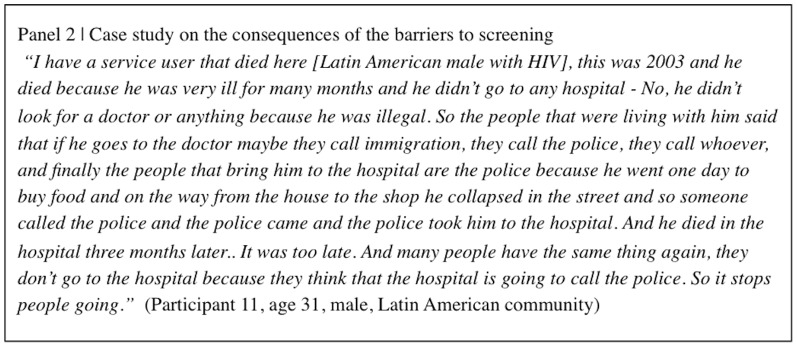
Case study on the consequences of the barriers to screening.

Participants highlighted a number of consequences that barriers can have for a new migrant patient and the wider community. Directly, the barriers stopped migrants from attending screening services as they “wait until their situation has got a little bit worse, when it's actually disabling them and they can't do any other activities” (Participant 14, age 40, male, African community) before they get tested for the diseases. “When they are screened later then the medication is not given the optimum chance to work for them…then obviously they have got a very narrow chance of recovery” (Participant 1, age 52, male, African community). Four participants mentioned that this “late diagnosis” has led to cases of death in their communities; a case study from one participant is presented in Figure 2.

Two participants mentioned that in the Eastern European community new migrants may turn to alternative forms of medication, for example, getting “herbs sent back from home” (Participant 2, age 44, male, Ukrainian community), “return back home” (Participant 7, age 31, female, Eastern European community) to do the screening or may also use services from individuals in the community “who they knew practiced medicine back in their own country” but who are not yet licensed in the UK (Participant 2, age 44, male, Ukrainian community). At the community level, participants raised concerns that by presenting late and not knowing ones status, new migrants with infections may be “putting others at risk” and increasing “the number of people being exposed to that risk” (Participant 6, age 34, female, Arabic speaking community).

### How do we improve access to screening?

Despite this lack of accessibility there was consensus among participants that screening is acceptable to new migrants, if services are promoted and offered in a tailored and sensitive manner, and uptake would be high if barriers were broken down. Participants collectively reported that between 50% and 100% of new migrants they represented would consider screening if it was more accessible to them. One participant mentioned that “in relation to their health they [the community] wouldn't hesitate to take part in the screening….because it's for their benefit.” (Participant 6, age 34, female, Arabic speaking community).

Key factors to consider when making screening more accessible included ensuring better collaboration between service providers and community organisations, as well as combining the screening of diseases into one appointment (table 3). Whether with faith-based or nationality based organisations, all participants strongly conveyed the message that screening services need to be taken out into the community and that the health service must work in partnership with community organisations. Community organisations are a key “asset” because they have the link to the migrant communities and hard-to-reach individuals (Participant 13, age 32, male, migrant communities). Interventions should be “co-owned” and based on the “principles of partnership about coexistence, collaboration, and cooperation” (Participant 13, age 32, male, all migrant communities) if screening services are to be made more accessible. Statutory service providers should focus on raising awareness of diseases, promotion of screening services, language and psycho-social support, as well as designing services that are migrant friendly and culturally sensitive with input from community organisations.

**Table 3:** Approaches identified to make screening more accessible.

Approach
Better access features
Build migrants confidence to access health services
Engage faith-based organisations
Ensure confidentiality
Improve hospitality and cultural sensitivity, through educating front-line staff on cultural competencies and migrant health needs
Increase language support
Increase psycho-social support
More appropriate and accessible promotion of screening
Offer one package of care for migrants, a general health check incorporating infectious disease screening
Outreach for isolated migrants
Raise awareness of diseases and screening in communities
Stronger collaboration with community organisations

Most of the participants argued that screening services need to be taken out of the hospital and into the community to make access easy, ”not sticking into one building or geographical location” (Participant 13, age 32, male, all migrant communities) and “delivering the service to the people where they are” (Participant 15, age 48, male, African community). Participants identified community settings such as places of worship, football games, community centre events, and carnivals as good opportunities to run screening interventions. As one participant explained, there is a bus outside the local market where - with the assistance of community health workers - individuals can “go in there, get fully screened, come back to the market and buy your goat meat and your plantain and go home knowing your status” (Participant 15, age 48, male, African community). Another participant made the point that people would prefer screening at the site of a community organisation, compared to a hospital, as they would feel more “comfortable” having attended the organisation before, and that these organisations have “staff employed from the community itself, when people come they may have a worker on hand who can talk them through the process in a culturally sensitive manner, or in a linguistically sensitive manner” (Participant 10, male, age 29, Asian community). By bringing screening into communities screening programmes will “work better and be far more effective” (Participant 15, age 48, male, African community).

15 of 20 participants considered that the best approach is to offer new migrants a package of care to include screening for TB, HIV, and hepatitis B and C at one appointment; the majority supported the idea of this being within a community setting. Participants suggested that screening be advertised as a general “health check” which would make migrants more receptive and considerably lessen the stigma of infectious diseases. Most importantly, participants felt that offering screening for multiple diseases at one appointment would lessen the dominant barrier of stigma that can prevent migrants from attending screening. This approach could be “immensely convenient for the person as well as the service” (Participant 15, age 48, male, African community). Participants suggested that packaging screening in this integrated way will reduce stigma, or “push stigma down” (Participant 13, age 32, male, all migrant communities) and participants would say yes to screening as it “is enough for people to take a minute and think okay, I know for a fact that I might not have HIV but I might well have the others” (Participant 6, age 34, female, Arabic speaking community).

## Discussion

This research highlighted strong agreement among health-care leads that screening for infectious diseases is currently inaccessible to the new migrant community in the UK. Interestingly, a key factor in poor uptake rate among the new migrant community was stigma and misconceptions that new migrant communities' themselves hold about the key diseases, deeply interlinked with a migrants' culture and faith, as well as perceived fears around the social implications of attending screening and receiving a positive diagnosis. Participants identified numerous barriers to accessing screening services at the current time - which were considered to be non-migrant friendly and culturally insensitive. New migrants are likely to be disproportionally affected by these barriers, and delays to diagnosis and treatment may have health consequences. However, there was strong consensus that acceptability of screening of the four key diseases is high among new migrants. Participants stressed the need to bring accessible and proactive screening into the community, strengthening collaborations with community-based organisations. They supported the idea of a community-based package of health screening combining all of the diseases into a general health check-up, with the aim of lessening the associated stigma.

We are aware that the views expressed by participants will reflect their own experiences of working with the health system around West London. While this may impact on the responses they provided, as community leads, their primary role is to represent their communities. We encouraged interviewees to talk about the wider communities around them; nevertheless, it is a challenge to have one group of people speak for another, and a separate study exploring the specific views of different groups of new migrants will be of interest. In addition, we are aware that a considerable number of participants were working in HIV as oppose to other infectious diseases under discussion, which will mean there may be an inevitable focus on barriers as they relate to HIV services.

Data are limited on the issue of infectious disease screening specifically in the new migrant community; however, numerous studies exist on the use of general health services by the wider migrant community and ethnic minority groups which confirm a myriad of potential barriers to access that confer with our findings [27]–[29]. Studies specifically exploring HIV testing barriers in migrants, including a systematic review [30] overlap with our findings in new migrants across all four diseases – including migrants having insufficient information about diseases and their prevention, lack of knowledge about health service provision, a perceived discriminatory attitude of health-care providers (including reception staff), fear of a lack of confidentiality and deportation, and confusion over entitlement to free health care [31]–[33]. Furthermore, studies on tuberculosis in “vulnerable groups” report that a key barrier to screening was to do with concerns around stigma within their own communities, and a fear of death [34]–[36]. For hepatitis B and C, previous studies in migrants and ethnic-minority groups report barriers related to language and culture, discrimination and stigma, low confidence in health services, lack of knowledge of available services, association of hepatitis testing with sexual health, and a low perception of disease risk [12], [37], [38]. What is clear is that the data themes we have documented are not unique to new migrants, but common experiences of migrant and ethnic minority groups affected by these diseases. However, it is our view that new migrants who are attempting to navigate a new health system and settle in a new community are likely to be disproportionally affected. That barriers to health care among new migrants may impact on health status has been previously reported [27], [33]. That acceptability for screening of HIV, TB, and hepatitis B and C is high among migrants has also been reported elsewhere, with migrants considered to be proactive about their health and screening “valued highly” [39], [40].

We found that there was a unanimous view among participants that to facilitate greater uptake of testing screening must be brought into the community, with service providers strengthening collaborations with community-based organisations. In the UK and elsewhere there are interesting examples of successful community outreach screening initiatives that target migrants for TB, HIV, or hepatitis (Table 4); however literature on community-based approaches are scarce with few high quality or controlled studies evaluating community models. We have found that data from innovative locally tested screening initiatives are often not published so the benefits of these approaches remain unclear. Conversely, international studies have reported unsuccessful community-based collaborations, in terms of uptake [41] and cost-effectiveness [42]. The Migrant-Friendly Hospitals Initiative, which resulted in The Amsterdam Declaration (Towards Migrant-Friendly Hospitals in an Ethno-culturally diverse Europe) in 2004, specifically calls for service providers to focus on developing partnerships with local community-based organisations with a view to improving service delivery to migrant groups [43]. Screening high risk groups for TB in General Practice/primary care has been formally assessed in a randomised controlled trial and found to be successful in terms of increased yield [44]. The optimum approach in high-migrant receiving countries is most likely to offer screening in a range of settings [39], [45], incorporating a strong focus on community engagement and partnership with migrant organisations in both the design and implementation of screening approaches.

**Table 4:** Examples of international models of community-based migrant screening collaborations for TB, HIV or hepatitis B and C.

Study	Model
Jafferbuoy et al. Scotland, UK [46]	Mosque and Islamic centre based screening
	Raised awareness and promoted screening for hepatitis B and C in the mosque. After raising awareness, community offered screening in the mosque one day a week.
	High uptake: 177/250 attendees coming forward for testing in the mosques
	Only a modest investment in staff time.
Sadler et al. London, UK [47]	Various community settings - bars, health promotion events, community centre and social gatherings
	Conducted a survey of sexual attitudes in addition to HIV test.
	High uptake: 94/114 took test
Lewis et al. UK [48]	Mosque based screening promotion
	Distributed 5000 viral hepatitis testing cards in Mosques, following an awareness campaign, encouraging viral hepatitis testing at GP surgery.
	Community awareness campaigns and leaflets do not directly lead to testing for viral hepatitis
Gany et al. New York, USA [49]	Airport holding lot
	Conducted TB counselling and screening for taxi drivers in the airport holding lot - drivers drove through the lane, placed their arms out for measurement of the tuberculin skin test reaction. 123 drivers who participated, two thirds (82) were at high risk for tuberculosis. Seventy-eight (63%) of the 123 returned for test readings.
Brassard et al. Montreal, Canada [50]	School-based screening
	Newly arrived immigrant children in selected schools were screened for latent TB. Family and household associates of the TST-positive child also were screened for LTBI. 542/2524 (21%) were TST-positive. Of 342 children started on therapy, 316 (92%) demonstrated adequate adherence. 599 associates investigated from the 484 TST-positive schoolchildren seen at the TB clinic. Of 555 associates with TST results, 211 (38%) were found to be TST-positive.
	Programme was effective and cost-effective.

The idea raised by participants of combining these diseases into some kind of general health check-up, with the aim of reducing the considerable stigma associated with infectious diseases, merits further exploration. Such an approach will need to be combined with awareness raising about the benefits of screening within new migrant communities, and attempts to facilitate high uptake to services, in an attempt to tackle misconceptions and reduce stigma. The UK's Health Protection Agency previously recommended that consideration be given to the idea of an extended New Patient Health Check for certain groups of migrants in primary care [6]. To what extent such an approach can be adopted in other high-migrant receiving countries is unknown, with countries taking a wide variety of approaches to screening for infectious diseases in this patient group [22]. Further research is now urgently needed to develop evidence-based community-focussed screening models - drawing on models of best practice and lessons learned from UK and internationally – as well as exploring how healthcare professionals can work more effectively with the new migrant community to facilitate improved access to screening.
